# Enhancing optical microscopy illumination to enable quantitative imaging

**DOI:** 10.1038/s41598-018-22561-w

**Published:** 2018-03-19

**Authors:** Emil Agocs, Ravi Kiran Attota

**Affiliations:** Engineering Physics Divison, PML, NIST, Gaithersburg, MD 20899 USA

## Abstract

There has been an increasing push to derive quantitative measurements using optical microscopes. While several aspects of microscopy have been identified to enhance quantitative imaging, non-uniform angular illumination asymmetry (ANILAS) across the field-of-view is an important factor that has been largely overlooked. Non-uniform ANILAS results in loss of imaging precision and can lead to, for example, less reliability in medical diagnoses. We use ANILAS maps to demonstrate that objective lens design, illumination wavelength and location of the aperture diaphragm are significant factors that contribute to illumination aberrations. To extract the best performance from an optical microscope, the combination of all these factors must be optimized for each objective lens. This requires the capability to optimally align the aperture diaphragm in the axial direction. Such optimization enhances the quantitative imaging accuracy of optical microscopes and can benefit applications in important areas such as biotechnology, optical metrology, and nanotechnology.

## Introduction

While optical microscopes have been used for research and analysis for a considerable period of time, calibration, quantitation, precision, and accuracy have only received major attention for the past decade or so^1–20^. Quantitative imaging in cell biology^1^, quantitative imaging for biomarker development^14^ and precision localization imaging for fluorescence microscopy^15–17^ are some prime examples of research areas where the importance of quantitative imaging has become well recognized. In biology, quantitative imaging refers to the extraction and use of numerical and statistical features from medical images. This process leads to an improved statistical and evidence-based approach to diagnosis and treatment^14^. Such numerical and statistical based approaches are also beneficial for non-medical image applications.

The quantitative imaging process includes standardization and optimization of image acquisition protocols. Several aspects of these protocols have been identified to minimize degradation of images in optical microscopy (including confocal and fluorescence microscopy). The list of these aspects includes the following: use of laser power, laser stability, uniform spatial illumination, flat-field correction, camera performance, optical aberrations, noise, benchmarking, colocalization, spectral registration, spectral reproducibility, lateral resolution, axial (*Z*) resolution, lens cleanliness, lens characteristics, temporal variability of signal and noise, one-step absolute intensity calibration, tool induced shift, calibration-on-spot, correcting field-dependent aberrations, and *Z*-drive reproducibility^1,4,18,20–26^. For example, field dependent aberrations can lead to a localization error of as large as 50–100 nm^26^.

Although uniform spatial intensity (obtained with a Kohler setup) has been cited as one of the more important aspects, achievement of uniform angular illumination symmetry at the sample plane is equally important to acquire consistently repeatable and reproducible quantitative values. Non-uniform angular illumination asymmetry across the field-of-view (FOV) has often been overlooked as a potential source of degradation for quantitative optical microscopy imaging. This asymmetry can be measured by using angular illumination asymmetry (ANILAS) maps^27–29^. The presence of ANILAS adversely affects image quality and alters intensity profiles even when the illumination intensity is spatially uniform at the sample plane. The variation in the intensity profiles can distort the numerical and statistical values extracted from the images and thus degrade quantitative imaging.

To the best of our knowledge, we demonstrate here for the first time that the ANILAS at the sample plane depends on objective lens design, illumination wavelength (λ), and the position of the aperture diaphragm (AD, sometimes also referred to as ‘condenser aperture diaphragm’ or ‘aperture stop’). To minimize the ANILAS across the field-of-view, one must optimize the combined selection of objective lens, λ, and AD location. Changes in any one of the three parameters, requires re-optimization of the other two. This means that nearly every objective lens type requires its own optimal alignment condition to enhance optical imaging precision and hence to obtain consistent quantitative values.

In the referenced works^27–29^, we proposed a simple and fast method to measure the ANILAS. A two-dimensional plot of the measured ANILAS values at the sample plane across the FOV results in an ANILAS map (Supplementary Fig. 1). An ANILAS map provides a convenient way to visualize imperfections in the angular illumination at the sample plane. A large ANILAS value in the map indicates a large angular illumination asymmetry (also referred to as either illumination imperfections, or distortions) resulting in image profile misrepresentation. To demonstrate the impact of ANILAS on image quality, we present the effect of illumination distortions on the optical images of isolated lines placed at various locations in the FOV in Supplementary Fig. 1^28^.

The presence of ANILAS not only affects optical images at the best focus, but also sometimes significantly affects through-focus images (see Supplementary Fig. 2)^29^. Distortions in the optical illumination could lead to quantitative misinterpretation of measurements that are based on through-focus images, such as confocal microscopy^30^, 3D super-resolution imaging of nanoparticles^31^, optical nanoparticle and bacteria tracking methods^32–36^, and the TSOM method^32,37–39^. Hence it is important to achieve lowest possible ANILAS values across the FOV for these applications as well.

**Figure 1:**
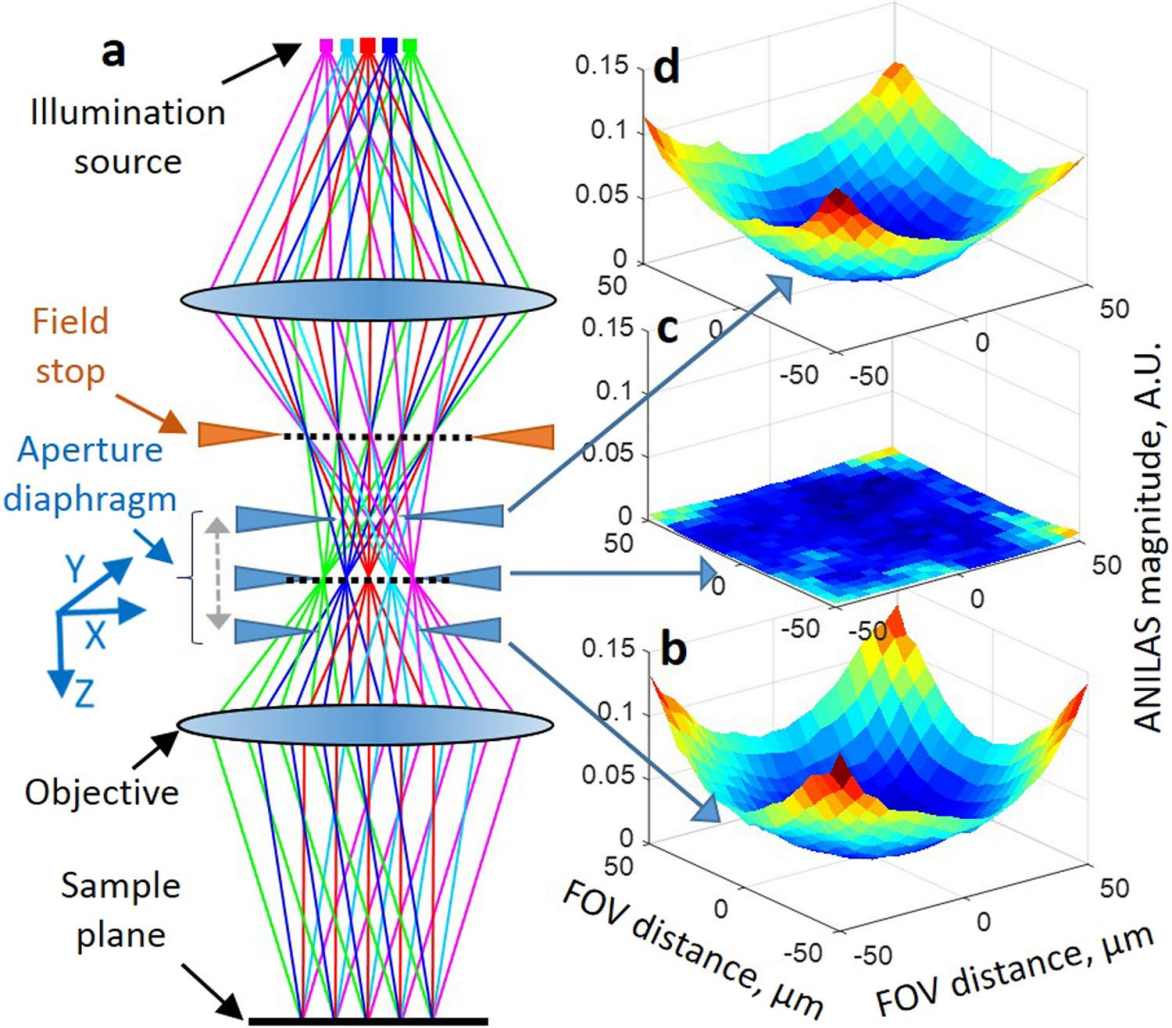
The effect of AD axial location on illumination distortions measured at the sample plane (**a**). Simplified schematic of a reflection (epi) type optical microscope. The AD (or stop) can be moved in the X, Y, and Z directions, as indicated. Measured ANILAS maps for objective type 1 (50×, NA = 0.55), with the AD at axial locations of (**b**) 2000 μm, (**c**) 500 μm and (**d**) −1500 μm. Which can be seen in the supplementary information Video S1. Animation showing changes in the ANILAS map as a function of the AD axial location. Schematic depiction of variations in the angular illumination at the sample plane is also presented at the bottom left.

In general, commercially available optical microscopes are well designed and aligned. However, these microscopes have the potential to readily lose their illumination optimization through misalignment of either the illumination source or the AD (Fig. 1a)^28^. In this paper, we study the effect of misalignment of the AD. But the same procedure can also be applied to analyze ANILAS due to illumination source mismatch. A lateral (*X-Y*) misalignment of the AD as small as a few tens of micrometers could introduce asymmetry in the angular illumination and lead to decreased image quality^28,29^. In our experience, this kind of misalignment is not uncommon, even in high-end microscopes. Microscopes that have adjustable ADs and illumination sources are likely to lose optimal lateral alignment over time. We have presented ANILAS maps for various non-optimal illumination conditions in a previous publication^28^. These studies revealed the importance of properly aligning the AD laterally to achieve optimal angularly-uniform illumination, even when all of the other optical elements were well aligned.

The axial position of the manufacturer-provided original AD is commonly fixed for optical microscopes. Here we show that the fixed axial position of the original AD could significantly deviate from the optimum axial location and sometimes result in unavoidable, inherent illumination distortions. To demonstrate this, we present here illumination analysis results (using the ANILAS maps) as a function of the axial position of the AD for each of several objective lens and illumination wavelength combinations. We also demonstrate how to achieve the optimal illumination conditions using the ANILAS maps.

## Results and Discussion

### The measurement process

A commercially available, research-grade optical microscope was used for the experiments described in^28^ with different magnification and numerical aperture (NA) objective types as given in Table 1. We used three LED illumination sources (along with band-pass filters) to produce narrow-band illumination centered around wavelengths of 405 nm, 520 nm and 633 nm, with a spread of approximately ±5 nm, ±5 nm, and ±2 nm respectively, at the full-width-half-max level. A nominal 400 μm fixed diameter aperture was used as the AD. The AD was mounted on an *X-Y-Z* translational stage so that its position could be aligned relative to the optical axis using micrometers (lateral (*X-Y*) with precision = ±2 μm, axial (*Z*) with precision = ±5 μm). ANILAS maps were evaluated by the method described in^28^ using an array of trenches in SiO_2_ with a nominal width of 100 nm and a pitch of 1000 nm over a Si substrate as a grating target. All ANILAS data reported here are averages of five repeats.

**Table 1:** Details of the objective types provided by the manufacturer that have been used. Obj. = objective, Mag. = objective magnification, FOV = field-of-view (the side of a square image captured), NA = objective numerical aperture. Higher number of stars for flatness and color correction indicates better quality objectives, with five stars being the highest rated objective.

Obj. Type	Obj. Design	Mag.	FOV (µm)	NA	Working Distance (mm)	Flatness	Color Correction
1	Plan Neo Fluar	50x	100	0.55	9.10	****	****
2	Plan	50x	100	0.70	1.10	**	***
3	Plan	50x	100	0.75	1.00	**	***
4	Plan Apochromat	50x	100	0.95	0.28	*****	*****
5	Plan	100x	50	0.80	1.00	**	**
6	Plan	100x	50	0.85	0.87	**	***
7	Plan	40x	120	0.60	2.20	**	***

Before starting the experiments, we carefully located the axial center position of the original AD provided by the microscope manufacturer with a measurement uncertainty of approximately ±100 μm. The original AD assembly was then removed and replaced with a 400 μm fixed diameter, custom aperture on the *X-Y-Z* micrometer stage such that its axial center coincided with the axial center of the original AD. This location was assigned a value of zero on the *Z*-axial coordinate. The AD was then moved 2000 μm away from the field diaphragm (in the positive *Z* direction). The ANILAS maps were evaluated at this axial location by adjusting the *X-Y* micrometer (lateral adjustment) such that the center minimum location of the ANILAS map coincided with the center of the FOV. This process was repeated by moving the AD axially toward the field diaphragm by 500 μm for each step until the axial position reached −1500 μm, for a total of 8 steps. The same procedure was repeated for each of the selected objectives shown in Table 1 and for an illumination wavelength of 520 nm. The procedure was repeated only for objective types 1, 4, and 6 for the illumination-wavelengths 405 nm and 633 nm. For the 633 nm illumination wavelength, a total of 9 steps were evaluated starting at 2500 μm away from the field diaphragm.

### The effect of axial location of the aperture diaphragm on the illumination distortions

Variation in the ANILAS map with AD axial position is shown in Fig. 1b–d, along with an animation of the same. We present the complete sets of ANILAS maps as a function of AD axial location for objective types 1, 4, and 6 and for the three illumination wavelengths in Supplementary Fig. 4.

**Figure 2:**
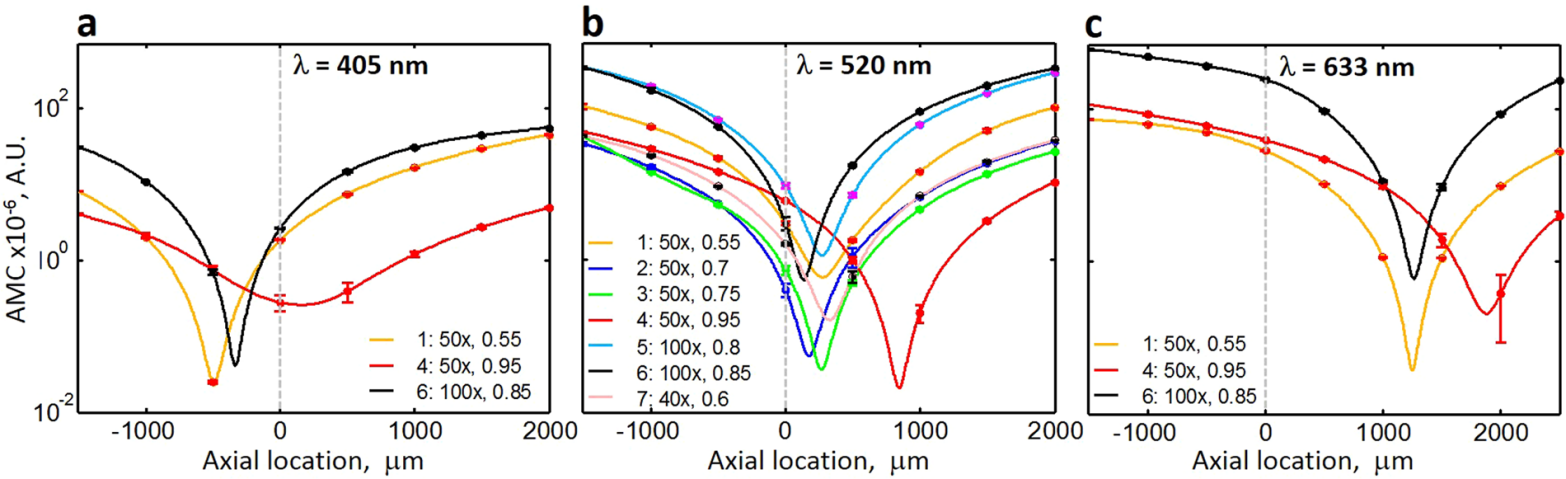
The relationship among objective type, wavelength, and the axial location of AD on the quality of illumination as characterized by ANILAS map curvature (AMC). The minimum AMC location represents the best quality illumination even though it deviates from the manufacturer-provided AD axial location. Plots are shown of AMC as a function of AD axial location for various objective types at illumination wavelengths of (**a**) 405 nm, (**b**) 520 nm, and (**c**) 633 nm. The objective type, magnification, and NA are shown in the legend in that order. Data points (filled circles) have been fitted with cubic spline curves. The dashed vertical line represents the original axial location of the AD. Error bars represent one standard deviation.

We can see that as the position of the AD deviates away axially from the nominal center position (Z = 0), the non-uniform illumination distortion (represented as curvature in the ANILAS maps) increases. We have quantified the illumination distortion by fitting quadratic surfaces to the ANILAS maps and extracting quadratic coefficient terms fitted separately along the X and Y axes for these surfaces. We have taken the average of these two quadratic coefficients to represent the mean curvature of the ANILAS maps, which we refer to here as ANILAS map curvature (AMC). Because the AMC is independent of the FOV, it is an appropriate measure for quantifying the ANILAS map. This measure is also convenient for the comparison of objectives with different magnifications and thus different sizes of the FOV. A plot of the AMC as a function of the axial position for all the objective types studied is shown in Fig. 2. The axial locations of the minimum AMC for all the objective types and wavelengths that have been studied in this paper are presented in Table 2.

**Table 2:** Minimum AMC axial positions for all the objective types and wavelengths studied in this paper along with their individual estimated standard measurement uncertainties.

Obj. Type	Mag.	Min. AMC Position (µm)			Meas. Uncertainty (µm)		
		For Wavelengths			For Wavelengths		
		405 nm	520 nm	633 nm	405 nm	520 nm	633 nm
1	50x	**−492**	273	**1252**	1.26	6.6	0.98
2	50x		170			21	
3	50x		269			8.5	
4	50x	**160**	**845**	**1874**	59	6.7	20
5	100x		273			4.9	
6	100x	−332	**135**	1262	2.2	3	1.7
7	40x		330			6.3	

The axial location where the minimum AMC occurs in Fig. 2 represents the best axial location of the AD in that it results in the lowest illumination distortion for that objective type, i.e., nearly uniform and symmetric angular illumination over the entire FOV, similar to Fig. 1c. Table 2 shows the axial location for minimal AMC for all the objective types and wavelengths studied in this paper. Any axial deviation of the AD to either side of this location results in an increased non-uniformity of illumination within the FOV. For example, see Fig. 1b,d. In addition, an AD properly centered in the lateral (X-Y) plane will result in the lowest illumination distortion at the center of the FOV, which coincides with the lowest point in the ANILAS map^28,29^. The illumination distortion increases as the distance from this central location increases towards the periphery of the FOV.

### Observations

A number of important observations can be made from Fig. 2. First, the axial AD location for the lowest distortion depends on the objective type even if all the other conditions are the same. In other words, every objective has its own optimum axial location. This implies that the one fixed axial AD location provided for most optical microscopes cannot physically match the optimum axial location for all objective types.

Second, the optimum axial location depends not only on the properties of the objective but also on the wavelength of the illumination. For the objectives tested, the location of the minimal AMC moves toward positive axial location as the wavelength of the illumination increases. For the microscope setup used here, Fig. 2b shows that the optimum axial location for objective type 6 (100x Plan, NA = 0.85) is the closest to the original fixed AD location (at approximately 135 μm) although it does not coincide with it. Hence for this objective, we can expect the best quality illumination and imaging using the original AD, but only for an illumination wavelength of 520 nm. Simply changing the illumination wavelength to 633 nm introduces significant illumination distortions for the same objective moving its minimal AMC location to 1262 μm. In addition, the AMC value becomes significantly higher (245 for the wavelength of 633 nm vs 2.97 for the wavelength of 520 nm) when the objective is positioned at the original AD axial location.

The third observation is that the degree of illumination distortion for any given axial location for a given objective increases as the displacement from its minimal AMC location increases. A corollary of this observation is that the illumination distortion for any given objective and wavelength at the original AD location increases as the distance from the minimum AMC location for that objective and wavelength to that original AD location increases. Therefore, under the current experimental conditions, the highest illumination distortion at Z = 0 can be expected for the illumination wavelength of 633 nm (see Fig. 2c). For the objectives and wavelengths tested, the range of minimum AMC axial distance is approximately 2366 μm (Table 2) implying that the AD should have approximately 2.4 mm axial range of motion so that it can be aligned for the best illumination under all the conditions studied. Unfortunately, the axial position of ADs is usually fixed preventing this alignment, and thus the majority of the optical microscopes have an inherent design limitation that may result in unavoidable illumination distortions.

**Figure 3:**
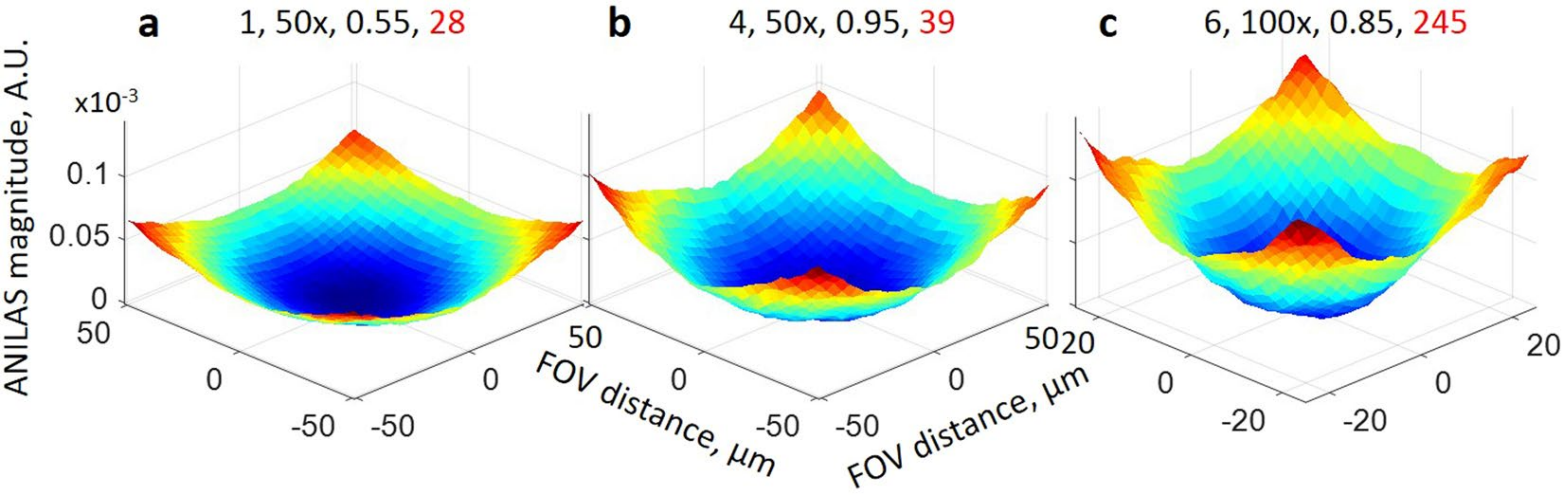
Illumination distortions as represented by the ANILAS maps with the AD located at the original axial location (Z = 0 um) for the illumination wavelength 633 nm. The objective type, magnification, NA, and AMC (x10^−6^) values are provided in the headers in that order. The FOV for (**a**) and (**b**) is approximately 100 μm, whereas for (**c**) it is approximately 50 μm.

Fourth and finally, the illumination distortions at the original axial location depend not only on the deviation of the minimum AMC location from the original axial location of the AD but also on the type of the objective. For example, for objective type 4 (50x, Plan Apochromat, NA = 0.95) the optimum axial location is displaced 1874 μm from the original AD location for the illumination wavelength 633 nm. For this objective, we can expect significant illumination distortions using the original AD. Even though the minimum AMC axial location for objective type 6 (100x Plan, NA = 0.85) is closer than that for objective type 4 (1262 μm vs 1874 μm), its AMC value is higher (Fig. 2c). Hence for objective type 6, we can expect even greater illumination distortions (located at the same distance from the center of the FOV) than those for objective type 4. For comparison, in Fig. 3 we have provided ANILAS maps at Z = 0 for the three objectives for the illumination wavelength 633 nm. Similar maps can be found in Supplementary Figs 5 and 6 for the other two wavelengths. Because the locations of the minimal AMCs generally deviate from the original axial location of the AD (Z = 0) in the current study, we can expect a systematic bias in quantitative measurements when one uses monochromatic illumination wavelengths.

### The selection of optimum illumination conditions

**Figure 4:**
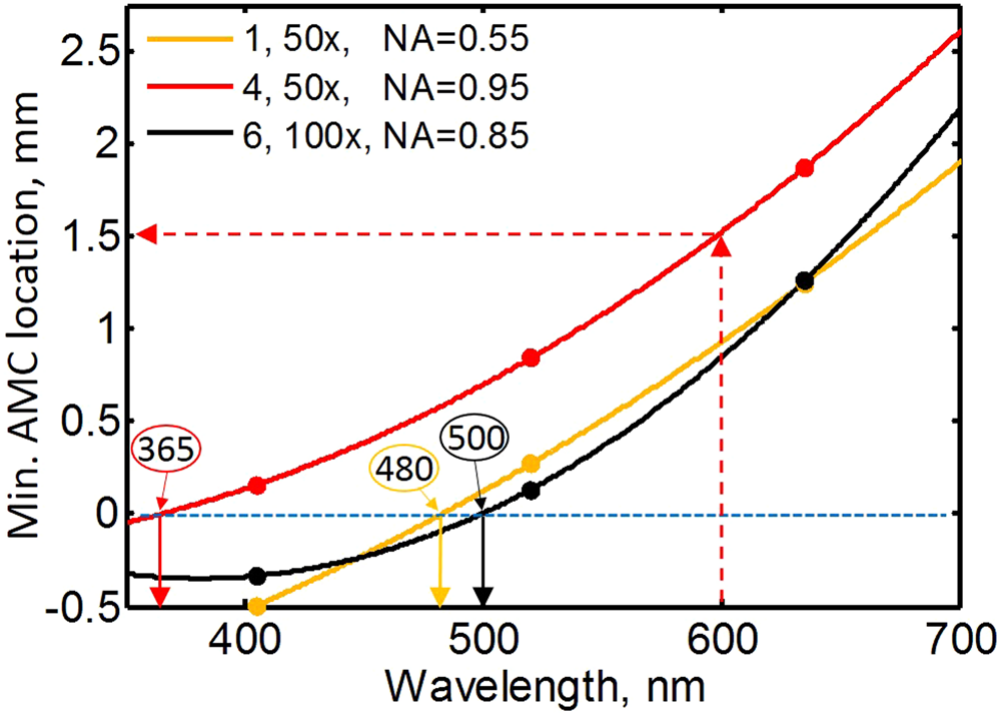
This figure shows how to select the optimum conditions to obtain illumination of the least distortion. The plot shows the AMC axial location for minimum distortion as a function of illumination wavelength for each of the three objectives. The horizontal blue dashed line shows the axial location of the original AD. Filled circles represent the measured data points. Quadratic curves were fitted to the data points. The solid arrows pointing down from the blue dashed line represent the approximate wavelengths at which the minimum AMC coincides with the original aperture axial location. The objective number, magnification and NA are shown in the legend in that order.

In Fig. 4, we recast and interpolate the minimum AMC location data shown in Fig. 2 as a function of wavelength for each of the three objectives to reveal additional useful information. Figure 4 enables determination of the wavelength for which a given objective produces the lowest illumination distortion when it is not possible to relocate the AD in the axial direction from its original position. We can determine that objective types of 1, 4 and 6 have the best illumination uniformity for illumination wavelengths of approximately 480 nm, 365 nm, and 500 nm, respectively. Conversely, if the AD location can be moved axially (to optimize for illumination), this figure allows determination of the optimal axial location of the AD for a given objective and wavelength to reduce distortion. For example, for objective type 4 and an illumination wavelength of 600 nm, an AD axial location of approximately 1.5 mm provides the best illumination condition (red dashed arrows). Figure 4 also shows that an AD axial alignment range of over 3 mm is required to achieve the best illumination condition for the visible spectrum and the objectives we have used.

### Demonstration of distortions in the image with illumination wavelength

As an example of the consequence of ANILAS, we here show the effect of the mismatch between the original diaphragm axial location and the minimum AMC axial location by looking at the resulting distortions in the data for the case of through-focus scanning optical microscopy (TSOM) imaging^37,40–43^. In an earlier publication^29^, we showed that a TSOM image central axis tilts (i.e., TSOM image distortion occurs) in the presence of an illumination distortion.

**Figure 5:**
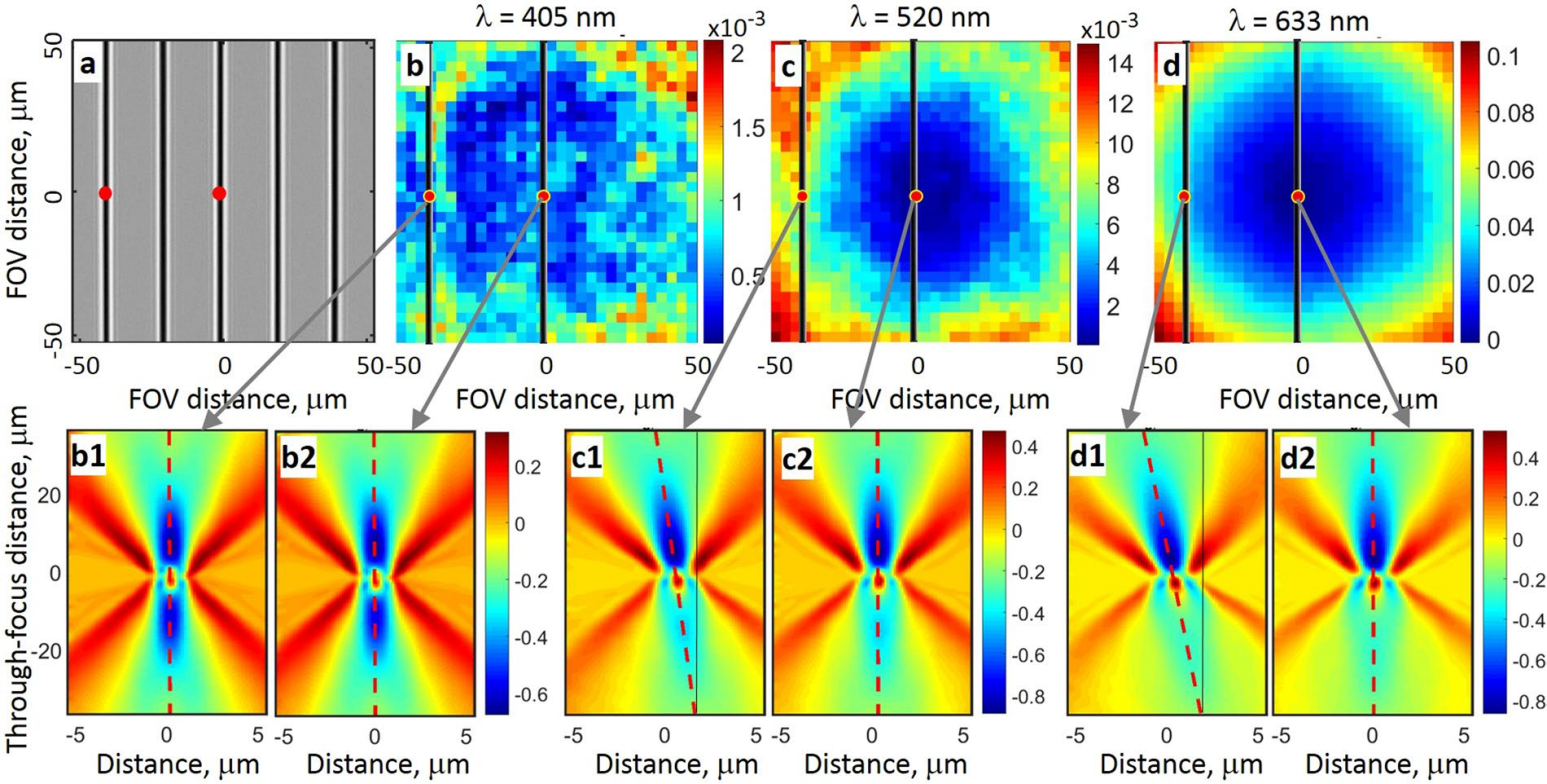
This figure demonstrates the detrimental effect of ANILAS caused by illumination variations upon the TSOM images. ANILAS maps and TSOM images are shown for objective type 4 (50x, NA = 0.95) with the AD located at the original axial position (*Z* = 0). (**a**) A typical optical image of the isolated Si line grating on Si substrate used to capture the TSOM images. Pitch is 20 μm, nominal line width is 1 μm, nominal line height is 100 nm. The TSOM images were captured at the locations indicated by the red dots. (**b**,**c**) and (**d**) are the measured ANILAS maps for the illumination wavelengths 405 nm, 520 nm, and 633 nm, respectively. The extracted TSOM image locations with respect to the ANILAS maps are also indicated by the red dots in (**b**), (**c**) and (**d**). (b1, b2), (c1, c2) and (d1, d2) are the captured TSOM images at the illumination wavelengths of 405 nm, 520 nm and 633 nm, respectively. The red dashed lines indicate the tilt of the TSOM image axis.

For the sake of argument, let us take the TSOM image axial tilt as the measurand and show how its location in the FOV and illumination wavelength affect its measurement when aberrations in the illumination are ignored or unknown. To demonstrate this, we selected an isolated line grating, shown in Fig. 5a, and measured the TSOM axial tilt at the two locations highlighted by the red dots in the image. The ANILAS maps measured with the aperture stop at the original axial location are shown in Fig. 5b–d for the three illumination wavelengths of 405 nm, 520 nm, and 633 nm, respectively. It can be observed that for the 405 nm illumination wavelength, the ANILAS map is relatively flat with low ANILAS values, indicating low illumination distortion. With this type of illumination, the TSOM images at the two selected locations in the FOV show nearly normal central axes (Fig. 5b1 and b2).

When the illumination wavelength is changed to 520 nm, the illumination is altered and the ANILAS map is no longer flat, as shown in Fig. 5c. If the same measurement were to be repeated at the 520 nm wavelength in the absence of this knowledge, it would produce a tilted TSOM image axis (Fig. 5c1) for the line located near the edge of the FOV. However, the same line located at the center of the FOV results in a near normal TSOM image tilt (Fig. 5c2) similar to that for the 405 nm illumination wavelength.

When the same test is repeated for the 633 nm illumination wavelength, there is even greater TSOM axis tilt (Fig. 5d1) for the line located near the edge, but again there is no tilt for the line located at the center of the FOV (Fig. 5d2). This means that the same target produces a different measurand based on its location within the FOV and the wavelength of the illumination. In the absence of the knowledge of the ANILAS, one would come to an erroneous conclusion based on the large variation in the observed tilt of the TSOM image axes. If the illumination is evaluated using the ANILAS maps, on the other hand, one would either minimize the illumination distortions by aligning the AD, or make an educated judgement based on the knowledge of the source of error in the measurand. In any case, the final conclusion would be more accurate.

**Figure 6:**
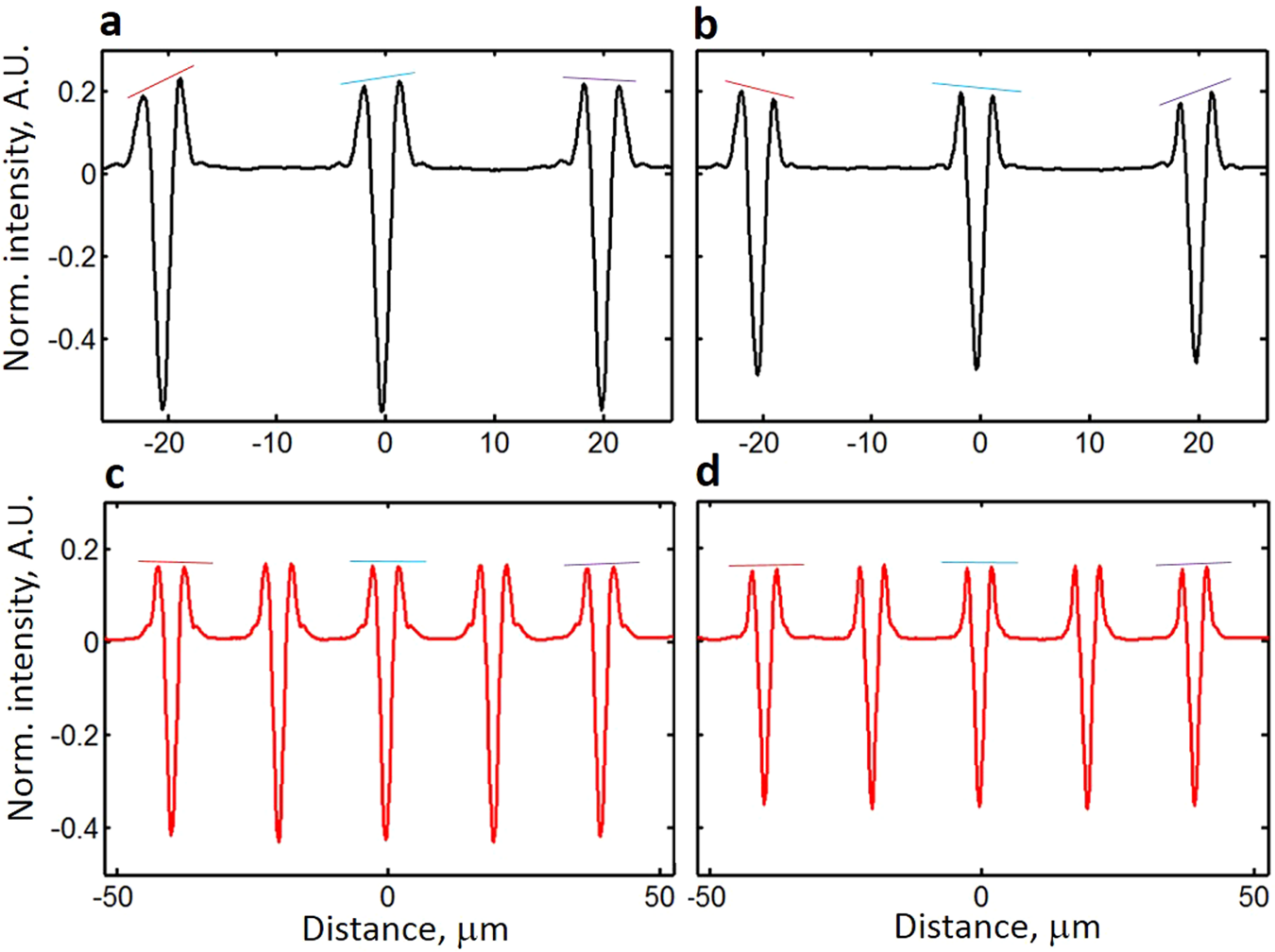
This figure demonstrates the detrimental effect of illumination distortions upon the intensity profiles. Shown here are variations in the intensity profiles within the FOV as well as the dependence of these variations upon the degree of illumination quality and upon the choice of focus position. The intensity profiles are for isolated Si line gratings on a Si background with nominally similar dimensions and symmetric cross sectional profiles (nominal line width is 1000 nm, nominal line height is 100 nm, pitch is 20 μm). An objective lens of type 6 (100x, NA = 0.85) was used with λ  = 633 nm for panels (a) and (b) while a type 4 (50x, NA = 0.95) was used with λ  = 405 nm for panels (c) and (d). The position of the AD was at the original AD axial location (Z = 0). Thus panels (a) and (b) were obtained with a strong ANILAS presence (Fig. 3c), whereas (c) and (d) were obtained using a good-quality illumination (Fig. 5b). Profiles in panels (a) and (c) were extracted by placing targets close to the position of best focus. Profiles in panels (b) and (d) were extracted by moving the targets axially 1.8 μm closer towards the objective than they were for panels (a) and (c). Optical intensities were normalized to simplify the comparison.

Here we present a second example that shows the importance of evaluating the angular illumination asymmetry. Because quantitative values are usually extracted from the intensity profiles of an image, we compare the intensity profiles of lines with nominally similar dimensions but located at different locations in the FOV to evaluate their profile differences. Objective type 6 produces a strong ANILAS and ANILAS non-uniformity across the FOV (Fig. 3c) when the AD is located in its original position, even though the illumination spatial intensity is uniform, indicating good Kohler illumination (Supplementary Fig. 7). Intensity profiles of the lines show significant variation with location in the FOV (Fig. 6a), including obvious differences at the peak heights. These variations are a direct consequence of the presence of ANILAS, as can be seen when compared with the intensity profiles obtained with objective type 4 with good illumination (Fig. 6c).

A second disturbing observation is that these profile differences also depend on the focus position. Moving the line target 1.8 μm away in the focus direction results in revised characteristics of the profile variability (Fig. 6b). This shows that in the presence of ANILAS, the profiles change based on the location in the FOV *and* on the focus position. As a result, the quantitative values that have been evaluated would exhibit considerable scatter making it harder to make a decision about, say, a medical diagnosis. However, with good quality illumination (see Fig. 5b), variation in the profiles is minimal within the FOV (Fig. 6c), and with focus positon (Fig. 6d), thus enhancing quantitative measurements.

The current study indicates that typical optical microscopes may have inherent design limitations that degrade the quality of illumination and affect imaging precision. As demonstrated above, the degree of illumination distortion varies with the type of objective and illumination wavelength used. Hence, for enhanced quantitative imaging precision, illumination optimization must be performed for a combination of illumination wavelength and the type of objective used. Changing either of them requires repetition of the optimization process.

The current study shows that microscopes must have the ability to align AD location both laterally and axially to achieve this. If optimization cannot be achieved, it is helpful to know the illumination imperfections in the optical system so that an educated judgment can be made on the basis of this information. For enhanced optical imaging precision, in addition to achieving uniform spatial intensity, it is important to also minimize ANILAS across the FOV. For applications where quantitative measurements are critical, such as for quantitative imaging, metrology and high precision applications, the presence of ANILAS certainly affects the results and hence should be minimized.

The current method of determining illumination distortions using ANILAS maps works for monochromatic illumination wavelengths. Hence the performance of microscopes using white light illumination has not been presented here. However, ANILAS illumination analysis using the TSOM method shows promise^29^ for all types of illumination wavelengths, including white light.

## Conclusions

Microscopes designed to achieve uniform spatial intensity (Kohler illumination) may not attain uniformly symmetric angular illumination at the sample plane. The presence of angularly asymmetric illumination represents an illumination imperfection that must be minimized for precision and quantitative imaging. It can result in distorted images both at the best focus position and along the through-focus axis.

ANILAS maps provide a convenient way to measure and visualize the quality of illumination at the sample plane. The quality of illumination was analyzed for a combination of several types of objective lenses and illumination wavelengths using the ANILAS maps obtained by scanning the location of the aperture diaphragm along the optical axis. We have demonstrated that the axial location corresponding to the lowest distorted illumination depends upon the objective lens type and the illumination wavelength. The spread of these optimal axial locations for the seven objectives and three wavelengths studied in this paper was about 2366 μm. Because the fixed axial location of the aperture diaphragm on most microscopes can differ significantly from these optimal axial locations, we expect a certain degree of angular illumination asymmetry and unavoidable illumination aberration for most objectives. To achieve high-quality optical images such as for precision or quantitative imaging, or for metrology applications, this illumination aberration must be minimized by aligning the aperture diaphragm with the best axial location for each objective and wavelength combination used. Hence, we propose to microscope manufacturers that they provide sufficient axial alignment capability for aperture diaphragms (in addition to lateral alignment capability).

Our future plans include extension of the material presented here to include investigations into illumination analysis for white light using the TSOM method.

## Methods

We have presented the theory and a detailed step-by-step procedure for ANILAS map evaluation method in earlier papers^27,28^. The same procedure has been used in the current ANILAS analysis, except that the optical intensity profiles of the grating target were smoothened using cubic-spline fit and interpolated with 7 points between any two data points (see Fig. 5 in ref.^28^).

### Statistical analysis

The estimated standard measurement uncertainty presented in Table 2 has been determined by dividing the standard deviation by the square root of the number of measurements.

### Data availability

Data that support the findings of this study are available from the corresponding author upon reasonable request.

## Electronic supplementary material

ANILAS animation

Supplementary information
