# Supplementary material for: Enhancing optical microscopy illumination to enable quantitative imaging
Source: Sci Rep. 2018 Mar 19;8:4782. doi: 10.1038/s41598-018-22561-w (PMC5859171; doi:10.1038/s41598-018-22561-w)
Supplement: Supplementary file 2 — Supplementary information [file 41598_2018_22561_MOESM2_ESM.pdf]

Supplementary information for:

Enhancing optical microscopy illumination to enable quantitative imaging

Emil Agocs and Ravi Kiran Attota*
Engineering Physics Davison, PML, NIST, Gaithersburg, MD 20899, USA
*corresponding author: ravikiran.attota@nist.gov

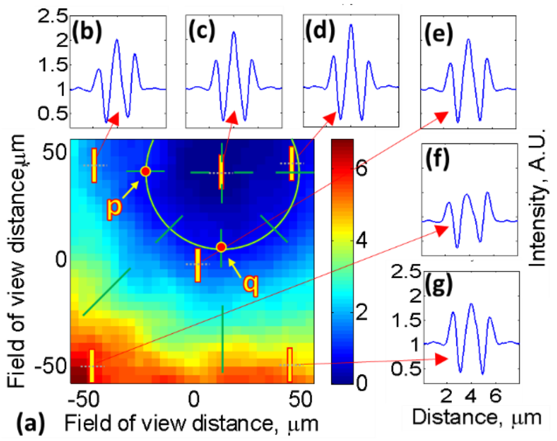

Supplementary Fig. 1. Variations in the optical intensity profiles as a function of location on the ANILAS map. (a) ANILAS map for the aperture stop location at (10 ± 2, 40 ± 2, -2000 ± 5) μm. (b), (c), (d), (e), (f), and (g) Intensity profiles of the same vertical line placed at different locations shown by the arrows. Dotted lines indicate the location of the extracted intensity profile. Reproduced from [28]

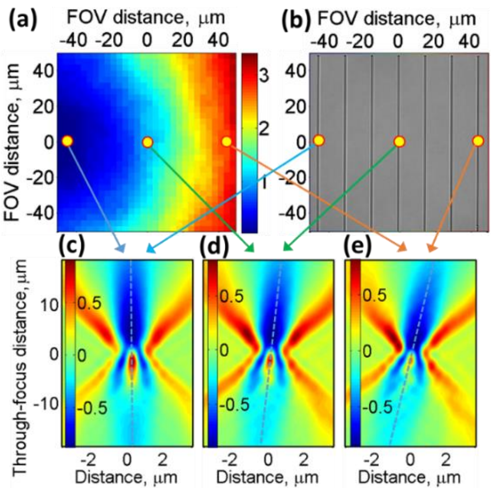

Supplementary Fig. 2. Comparison of ANILAS map and slant-TSOM images. (a) ANILAS map obtained by moving the AD axially toward the field diaphragm and laterally to the left (X = (-50 ± 2) μm, Y = 0, Z = (1250 ± 5) μm). (b) An optical image of several vertical line targets obtained under the illumination condition shown in (a). TSOM images (c), (d), and (e) are of the line targets denoted by arrows from (b). These locations within the microscope field are also indicated by arrows from (a). (The nominal line widths in (c), (d) and (e) are 950 nm, 800 nm, and 650 nm, respectively). Reproduced from [29].

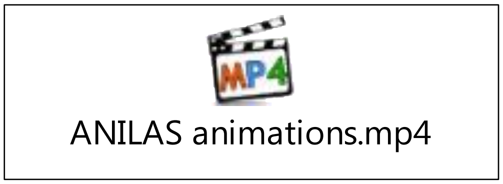

Supplementary Fig. 3. Animation showing changes in the ANILAS map as a function of the AD axial location. Schematic depiction of variations in the angular illumination at the sample plane is also presented at the bottom left.

Supplementary Fig. 4a.

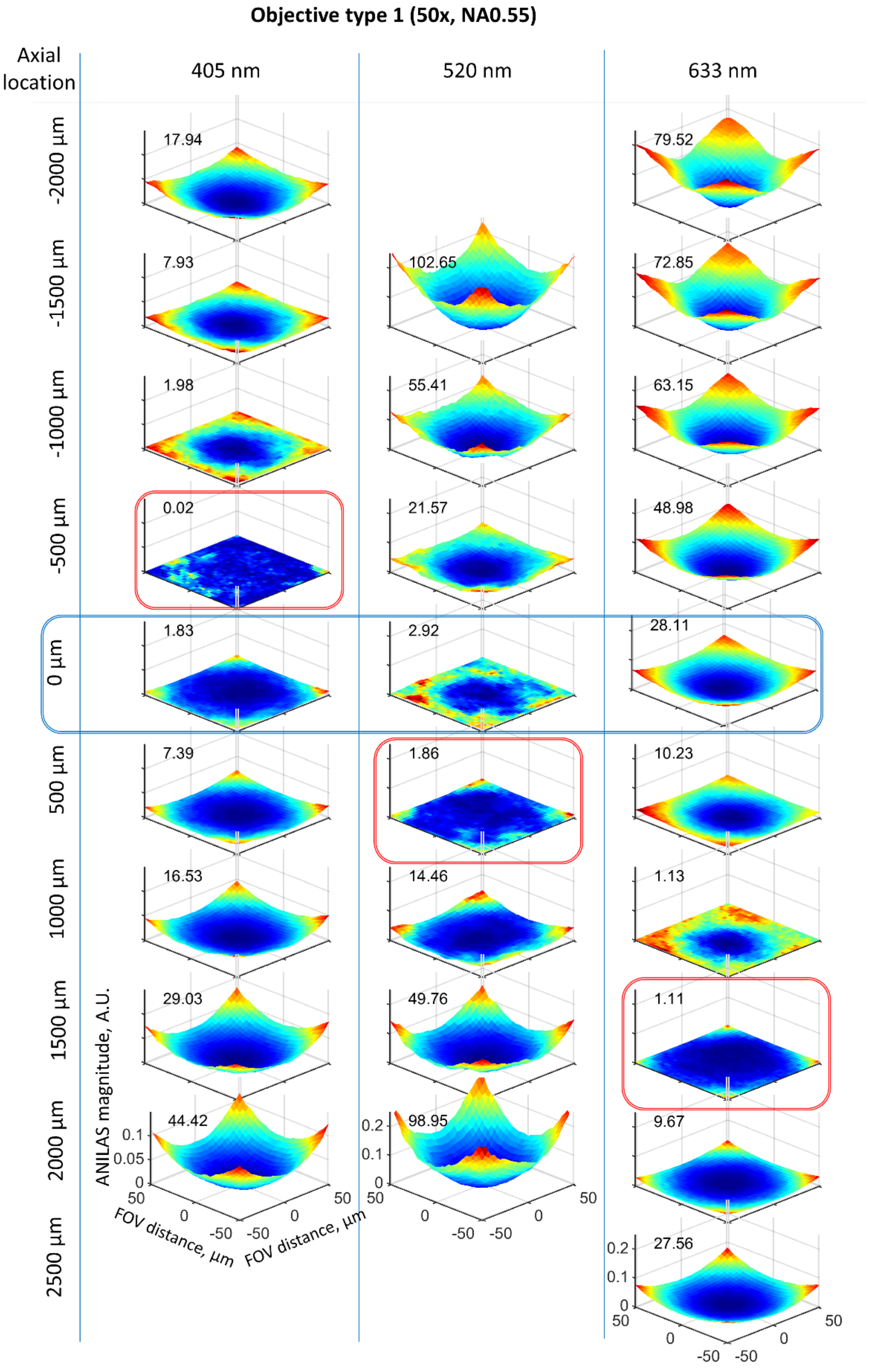

Supplementary Fig. 4b.

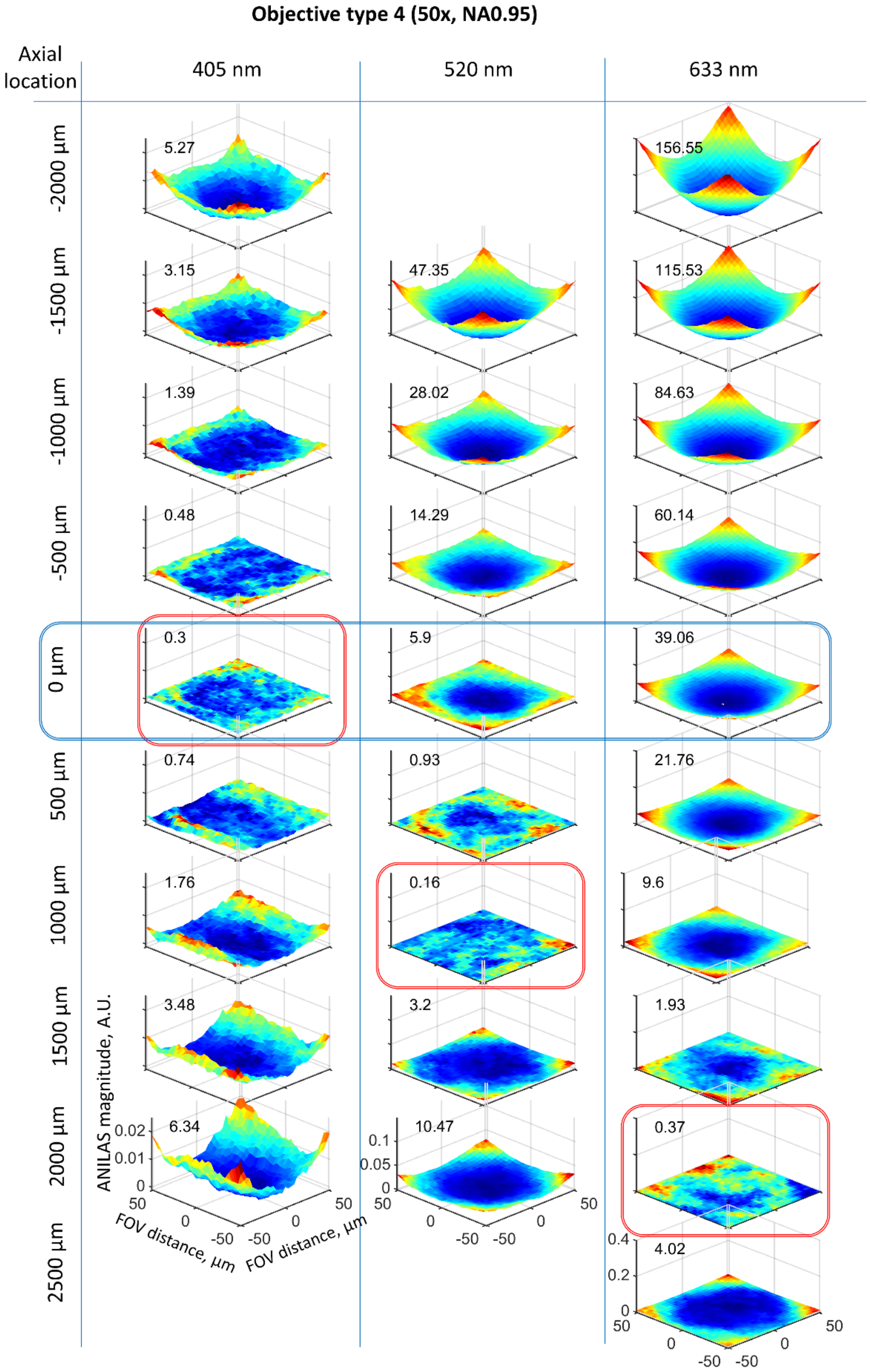

Supplementary Fig. 4c.

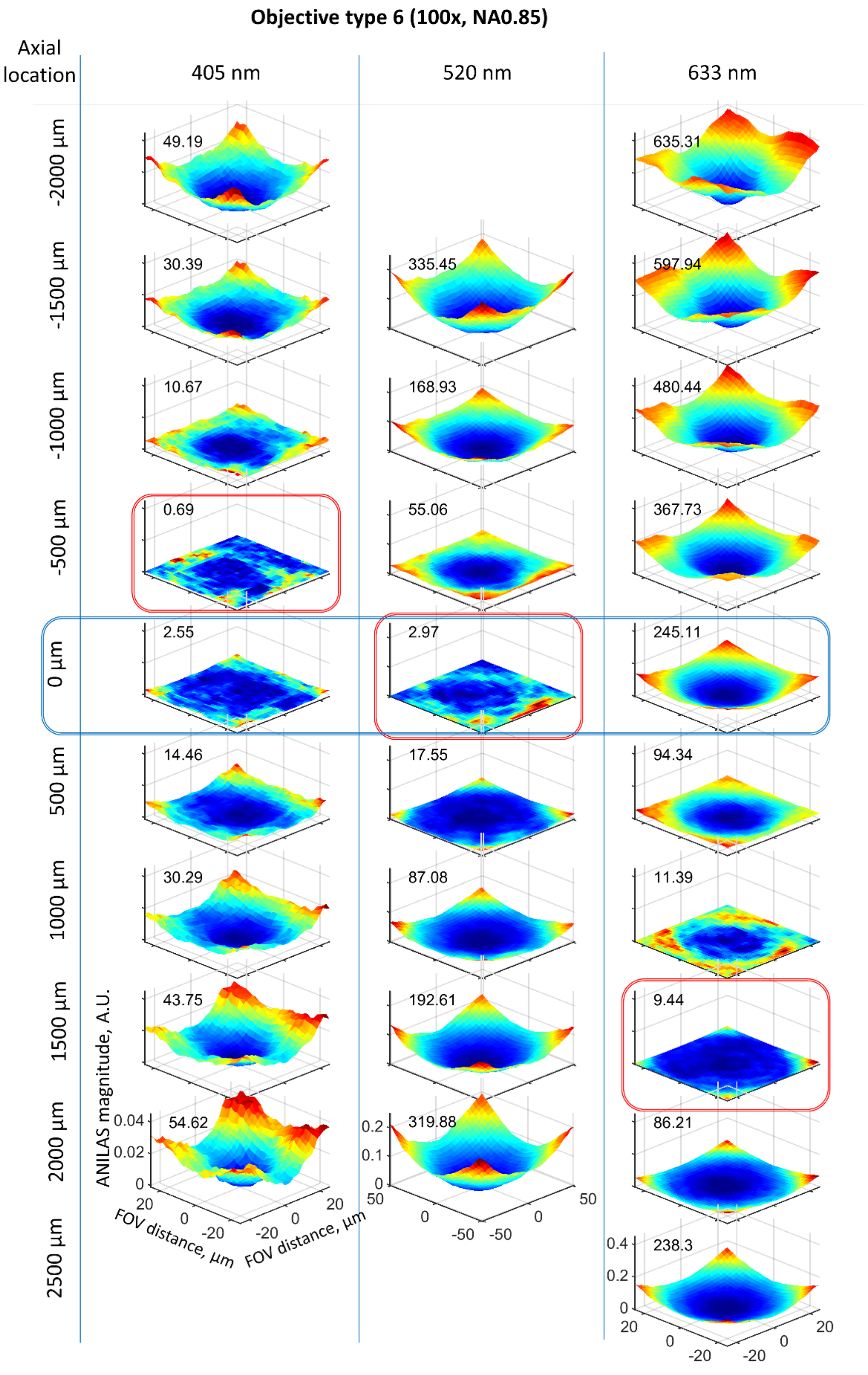

Supplementary Fig. 4. The effect of the AD axial location on the ANILAS maps. Variations in the ANILAS maps for the objective types (a) 1, (b) 4, and (c) 6 as functions of the wavelength and the AD axial location. AMC ($x10^{-6}$) magnitude is shown in the header for each ANILAS map. The blue boxes represent the axial location of the original AD, and the red boxes represent the ANILAS map closest to the minimum AMC location.

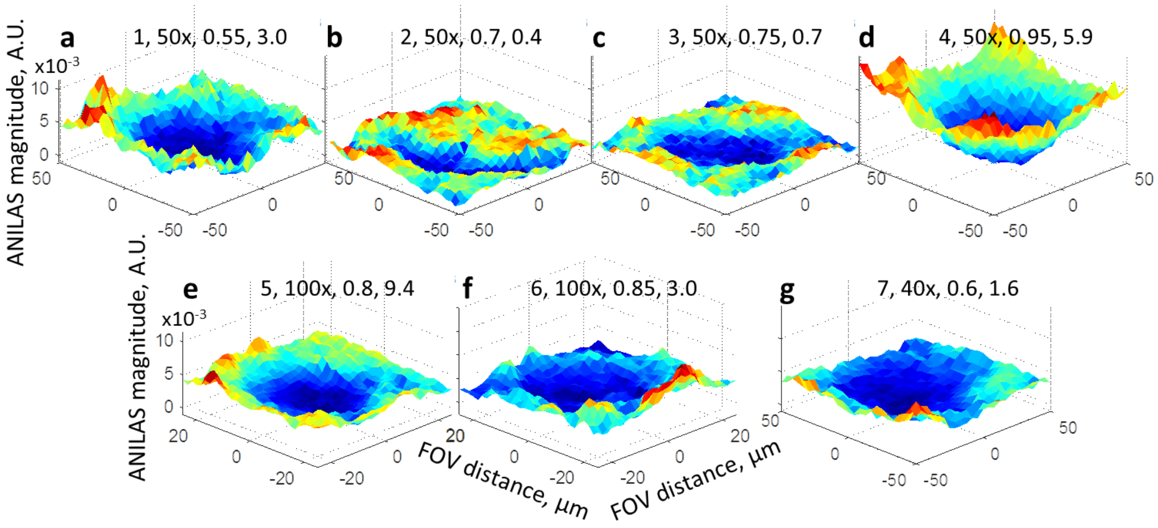

Supplementary Fig. 5. Variations in the ANILAS maps for the seven objectives with the AD located at the original axial location (Z = 0) for an illumination wavelength of 520 nm. The objective type, magnification, NA and AMC ($x10^{-6}$) values are provided in the headers in the same order.

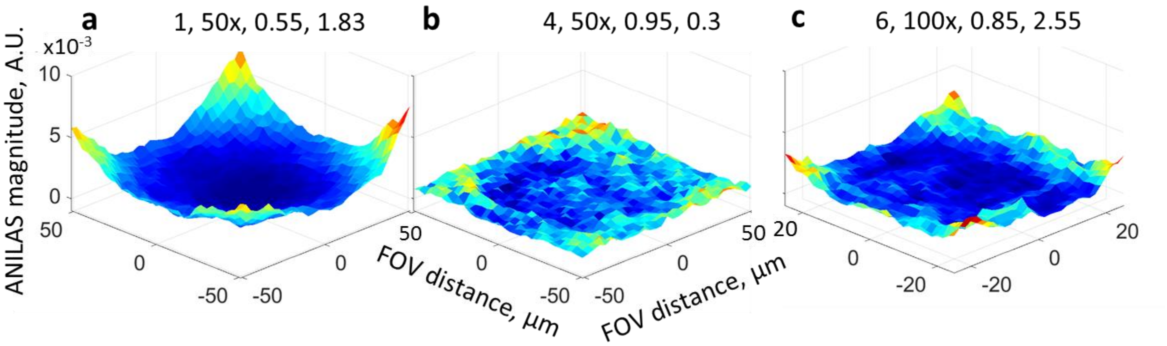

Supplementary Fig. 6. Variations in the ANILAS maps with the AD located at the original axial location (Z = 0) for an illumination wavelength of 405 nm. The objective type, magnification, NA and AMC ($x10^{-6}$) values are provided in the headers in the same order.

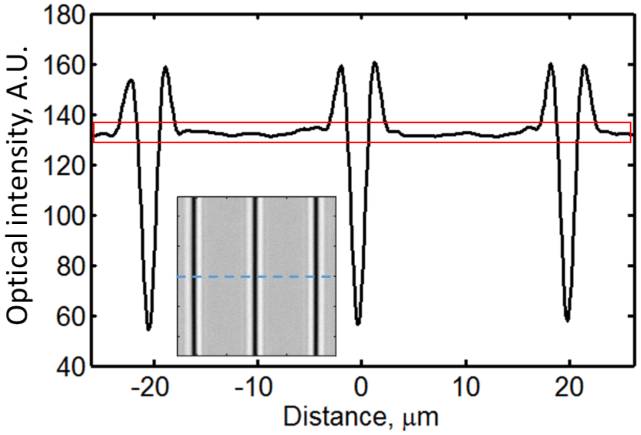

Supplementary Fig. 7. Raw intensity profile showing fairly uniform spatial intensity across the FOV. Raw profile extracted across the center of the FOV (along the dotted line shown in the inset) shows uniform spatial intensity as highlighted by the red box, even though the ANILAS map indicates the presence of strong illumination aberrations (Fig. 3c). Objective type = 6 (100x, NA = 0.85), λ = 633 nm, the AD at the original axial location (Z = 0), Si lines on Si background, nominal line width = 1000 nm, nominal line height = 100 nm, pitch = 20 μm.
